# SSRP1 Is a Prognostic Biomarker Correlated with CD8^+^ T Cell Infiltration in Hepatocellular Carcinoma (HCC)

**DOI:** 10.1155/2021/9409836

**Published:** 2021-02-23

**Authors:** Guanshui Luo, Jianguo Xu, Zhenglin Xia, Shuai Liu, Hong Liu, Ke He, Guoan Xiang

**Affiliations:** ^1^Department of Postgraduate Studies, The Second Clinical College of Southern Medical University, Guangzhou, Guangdong 510515, China; ^2^Department of General Surgery, Guangdong Second Provincial General Hospital, Guangzhou, Guangdong 510317, China; ^3^Department of General Surgery, Heyuan People's Hospital, Heyuan, Guangdong 517000, China

## Abstract

**Background:**

Hepatocellular carcinoma (HCC), one of the most common primary malignancies, is theoretically an epitope candidate for immune checkpoint inhibitors, and therefore, the identification of HCC biomarkers is important. Structure-specific recognition protein 1 (SSRP1) is involved in almost all chromatin-related processes, including DNA replication, repair, and transcription. However, its role in HCC remains to be elucidated.

**Methods:**

This study investigated the expression of SSRP1 in HCCDB, Oncomine, HPA, and other databases. The prognostic value of SSRP1 in HCC and its relationship with clinical characteristics were then explored using Kaplan-Meier plotter. At the same time, SSRP1 coexpression genes were explored and functionally annotated in the LinkedOmics database. Finally, the correlation between the SSRP1 expression and HCC immune cell infiltration was explored in TIMER and online single-cell sequencing database.

**Results:**

Significantly elevated transcriptional and proteomic SSRP1 expressions were found in HCC. Increased SSRP1 mRNA expression was significantly correlated with relevant clinicopathological parameters such as immune cells. Notably, the SSRP1 expression was positively correlated with the infiltration levels of Treg and CD8^+^ T cells, especially exhausted CD8^+^ T cells. Interestingly, the SSRP1 expression was higher in both tumor Treg and exhausted CD8^+^ T cells than in adjacent tissues.

**Conclusion:**

SSRP1, as a new prognostic marker for HCC, promotes HCC development by influencing the infiltration of depleted CD8^+^ T cells and may influence the effect of immunotherapy.

## 1. Introduction

HCC is a common malignant tumor. It is reported that the incidence of liver cancer continues to increase in recent years, the 5-year relative survival rate is low, and the mortality rate is increasing [1]. At present, the treatment strategy used in clinical practice is still routine surgery, supplemented by interventional therapy, radiotherapy, and chemotherapy. However, the effect of these treatments is limited, and the recurrence rate is high. The latest research on immunotherapy shows that it is the hope of overcoming cancer in the future [2].The tumor microenvironment coexists and interacts with various immune cells to maintain the growth of liver cancer [3]. The tumor microenvironment of HCC is characterized by the dysfunction of the immune system through a variety of mechanisms, including the interaction between immune checkpoint ligands and receptors [4]. Based on the increased expression of PD-L1 and the increase of tumor-infiltrating lymphocytes (TILs), HCC is theoretically an eximious candidate for immune checkpoint suppression [5]. Therefore, there is an urgent requirement to clarify the immunophenotype of tumor-immune interaction in hepatocellular carcinoma and to identify new immune-related therapeutic targets.

SSRP1 is a subunit of histone chaperone that facilitates chromatin transcription (FACT) and is involved in almost all chromatin-related processes, including DNA replication, repair, and transcription [6–10]. In terms of mechanism, research has shown that FACT plays an important role in promoting the transition of cancer cells from G0 to the proliferative state [11]. Clinically, the increased expression of SSRP1 in breast cancer is related to known markers of poor prognosis [12]. In HCC, the expression of SSRP1 is upregulated and promotes the occurrence and development of HCC [13]. In addition, SSRP1 inhibitor Curaxin CBL0137 has shown anticancer effects in many preclinical cancer models and is currently in phase I clinical trials targeting solid tumors and hematological malignancies in adults [14]. In glioblastoma (GBM), CBL0137 activates p53 and inhibits NF-*κ*B transcription [15]. Similarly, CBL0137 effectively inhibits the growth of HCC and makes HCC cells sensitive to sorafenib [16]. It has been shown that SSRP1 plays an important role in the development and progression of HCC. Although previous studies on SSRP1 have focused on chromatin-related aspects, this study will explore the potential function of the SSRP1 expression in tumor immunology.

This study comprehensively analyzed the expression of SSRP1 in HCCDB, Oncomine, and HPA databases. Then, we analyzed the value of the SSRP1 expression in the prognosis of HCC patients. In addition, we investigated the correlation between SSRP1 and tumor-infiltrating immune cells in different tumor microenvironments by TIMER, online hepatocellular single cell sequencing, and other databases. This study elucidates the important role of SSRP1 in HCC and provides potential relationships and mechanisms for the interaction of SSRP1 with tumor immunity.

## 2. Materials and Methods

### 2.1. HCCDB Database

HCCDB is a database of the HCC expression atlas containing 15 public HCC gene expression datasets containing totally 3917 samples [17], including the data from the Gene Expression Omnibus (GEO), Liver Hepatocellular Carcinoma Project of The Cancer Genome Atlas (TCGA-LIHC) and Liver Cancer-RIKEN, and JP Project from International Cancer Genome Consortium (ICGC LIRI-JP). The database is freely accessible at http://lifeome.net/database/hccdb.

### 2.2. Oncomine Database

The expression level of the SSRP1 gene in liver cancers was examined in the Oncomine database (https://www.oncomine.org/). Oncomine is a cancer microarray database and web-based data-mining platform. The threshold was determined according to the following values: *p* value = 0.01, fold change = 1.5, gene rank = 10%, and data type: mRNA.

### 2.3. The Human Protein Atlas Database

The Human Protein Atlas (https://www.proteinatlas.org) is a Swedish-based program initiated in 2003 to map all the human proteins in cells, tissues, and organs using integration of various omic technologies [18]. Staining intensity, quantity, location, and patients' information in patients with the respective cancer types were available online. In this study, representative protein expressions of IHC images of SSRP1 were detected in HCC and normal tissues in Human Protein Atlas.

### 2.4. The Kaplan-Meier Plotter Database

The Kaplan-Meier plotter (http://kmplot.com) is capable to assess the effect of 54 k genes on survival in 21 cancer types. The system includes gene chip and RNA-seq data sources for the databases that include GEO, EGA, and TCGA [19]. The Kaplan-Meier plotter was used to analyze the relationship between the SSRP1 expression and overall survival time (OS) and progression-free survival time (PFS) in liver cancer data, and log-rank *p* and HR values were obtained simultaneously.

### 2.5. LinkedOmics Database

The LinkedOmics database (http://www.linkedomics. org/login.php) is a web-based platform for analyzing 32 TCGA cancer-associated multidimensional datasets [20]. The SSRP1 coexpression was analyzed statistically using Spearman correlation coefficient. Function module of LinkedOmics performs analysis of the Gene Ontology biological process (GO_BP) by the gene set enrichment analysis (GSEA). The threshold was determined according to the following values: *p* value, minimum number of genes = 5, and simulations = 1000.

### 2.6. TIMER2.0 Database

TIMER is a comprehensive resource for systematical analysis of immune infiltrates across diverse cancer types. This version of webserver (http://timer.cistrome.org/) provides immune infiltrates' abundances estimated by multiple immune deconvolution methods [21]. We analyzed the SSRP1 expression in LIHC and the correlation of the SSRP1 expression with the abundance of immune infiltrates, including Tregs and CD8^+^ T cells.

### 2.7. Online Single Cell Sequencing Database

Zhang et.al combined full-length and 30scRNA-seq technologies to obtain high-quality data for a large collection of CD45^+^immune cells from various tissues in HCC patients. Analysis of such HCC data can also be found at http://cancer-pku.cn:3838/HCC [22]. In the present study, we analyzed in this database not only the proportion of different types of CD8^+^ T cells in tumors and adjacent tissues but also the SSRP1 expression in Tregs and exhausted CD8^+^ T cell in tumors and adjacent tissues.

### 2.8. Statistical Analysis

The Kaplan-Meier plotter was used to assess how the SSRP1 expression and other pathological and clinical factors (stage, CD4^+^ T cells, CD8^+^ T cells, etc.) affect OS. *p* values <0.05 were considered statistically significant.

## 3. Result

### 3.1. The Elevated Expression of SSRP1 in HCC

**Figure 1:**
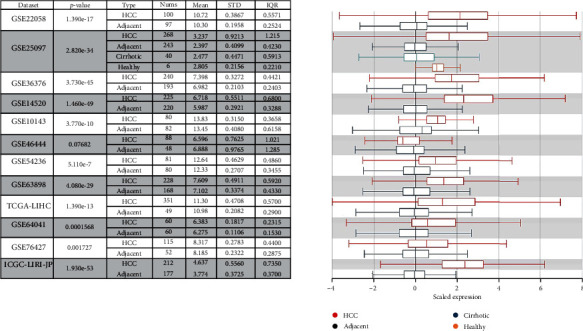
Chart and plot showing the expression of SSRP1 in tumor tissues and the adjacent normal tissues, according to *t*-test in HCCDB.

**Figure 2:**
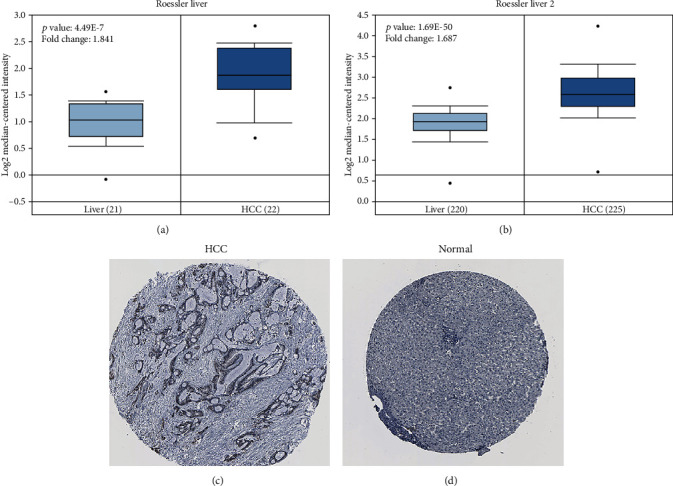
Expression of SSRP1 at transcriptome and protein levels. (a, b) Box plot showing SSRP1 mRNA levels in the Roessler Liver and Roessler Liver 2, respectively. (c, d) The expression of the SSRP1 protein in the normal liver tissue and HCC was visualized using immunohistochemistry via HPA.

Initially, we explored SSRP1 transcript levels in TCGA and multiple GEO databases. In the HCCDB database, analysis of 13 HCC cohorts consistently showed that the SSRP1 mRNA expression was significantly higher in HCC tissues than in adjacent normal tissues (Figure 1). Similarly, data from the Oncomine database indicated that the SSRP1 mRNA expression ranked within the top 10% (Figures 2(a) and 2(b)). In addition, the HPA data showed that SSRP1 staining was not detected in normal liver tissues, whereas low levels of expression were observed in HCC tumor tissues (Figures 2(c) and 2(d)). Taken together, these results suggest that SSRP1 is highly expressed at the transcriptional and proteomic levels in HCC tissues compared to normal tissues.

### 3.2. The SSRP1 Expression Is Survival-Associated

**Figure 3:**
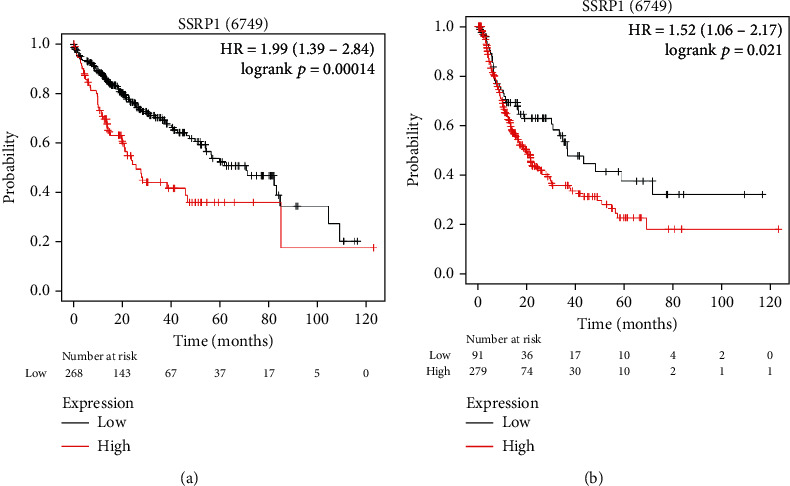
SSRP1 is associated with survival outcome. (a, b) Overall survival (OS) and disease-free survival (DFS) in TCGA LIHC cohort. The numbers below the figures denote the number of patients at risk in each group.

The relationship between the SSRP1 expression and survival outcomes in HCC was subsequently explored by using the Kaplan-Meier plotter. The database was divided into two groups based on SSRP1 expression levels. In LIHC, overall survival (OS) (log-rank test, *p* < 0.05) and progression-free survival (PFS) (log-rank test, *p* < 0.05) were significantly shorter in the SSRP1 high expression group compared to the low expression group (Figures 3(a) and 3(b) ). Therefore, it is conceivable that the high SSRP1 expression is a risk factor and contributes to the poor prognosis of HCC patients.

### 3.3. The High Expression of SSRP1 Affects Prognosis of Liver Cancer in Patients with Immune Cell Infiltration

To better understand the relevance and underlying mechanisms of the SSRP1 expression in cancer, we examined the relationship between the SSRP1 expression and clinical characteristics of liver cancer patients in the Kaplan-Meier plotter database. Specifically, the high SSRP1 mRNA expression was associated with worse OS of CD8^+^ T cell (HR = 3.05, *p* < 0.01) and Treg (HR = 1.75, *p* < 0.01) infiltration in hepatocellular carcinoma patients (Table 1). These results suggest that the SSRP1 expression levels can influence the prognosis of hepatocellular carcinoma patients with immune cell infiltration.

### 3.4. Functional Annotations

**Figure 4:**
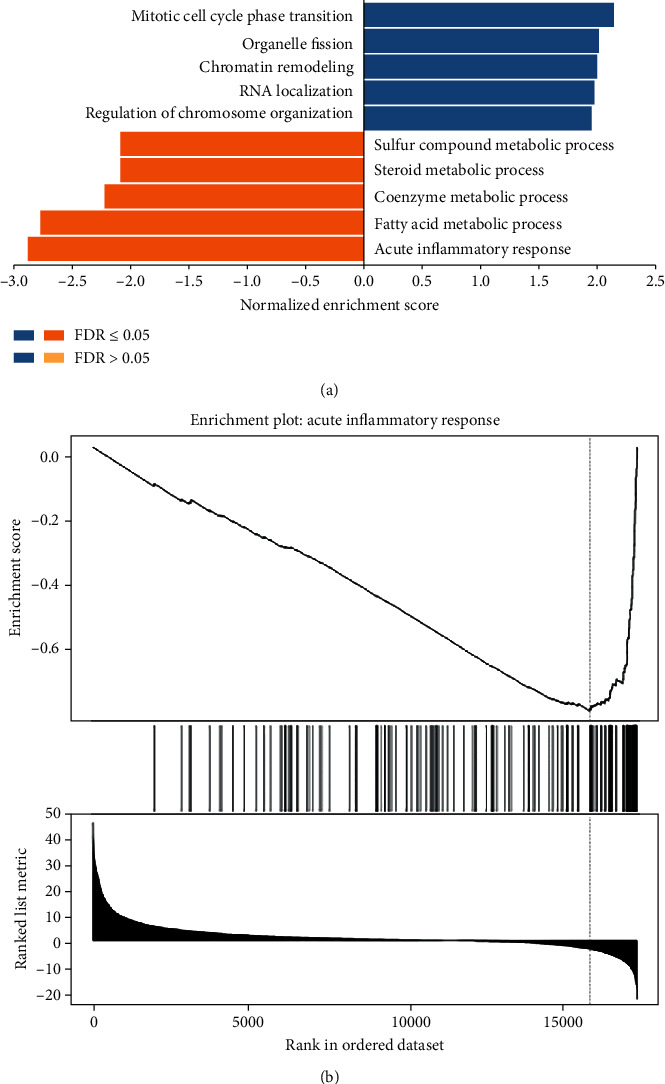
Functional annotations. (a)Significantly enriched GO annotations of SSRP1 in LIHC cohort (LinkedOmics). (b) One of the most important pathways involving the most negatively associated genes is the acute inflammatory response.

To further investigate the biological significance of SSRP1 in HCC, the coexpression pattern of SSRP1 was explored in the LinkedOmics database. Gene set enrichment analysis (GSEA) annotation of significant gene ontology (GO) terms revealed that SSRP1 coexpressed genes are mainly involved in DNA replication, chromosome segregation, and cell cycle G2/M phase transition, while acute inflammatory response, peroxisome organization, and lipolysis processes are suppressed (Figure 4). The above results imply that SSRP1 is involved in the immune response and inflammatory response.

### 3.5. Correlation of SSRP1 and Immune Infiltration Level

**Figure 5:**
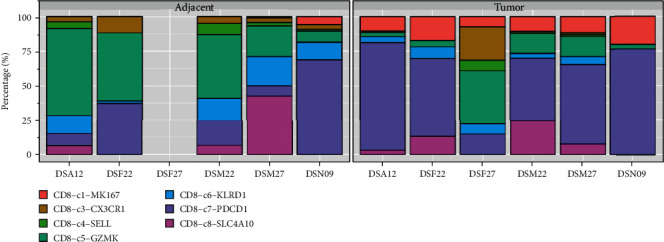
Percentage of different cell types in tumors and adjacent tissues of the same patients. CD8-C1-MK167: proliferative CD8^+^ T cells; CD8-C7-PDCD1: exhausted CD8^+^ T cells.

**Figure 6:**
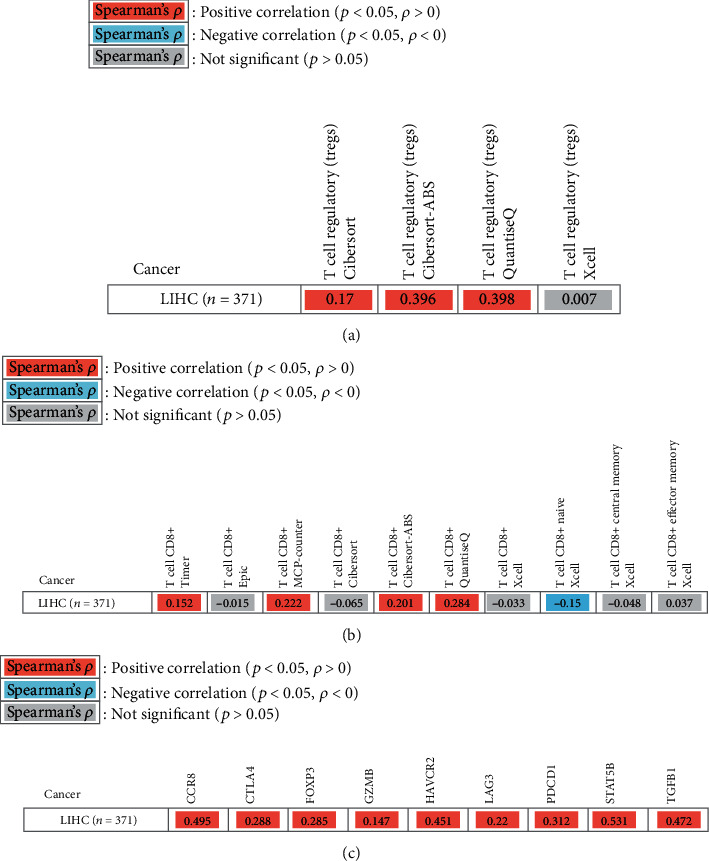
Correlation of the SSRP1 expression with TILs and immune marker genes. (a) Correlation of the SSRP1 expression with Tregs. (b) Correlation of the SSRP1 expression with CD8^+^ T cells. (c) Correlation of the SSRP1 expression with immune marker genes. CCR8: chemokine receptor 8; CTLA4: cytotoxic T-lymphocyte-associated protein 4; FOXP3: forkhead box P3; GZMB: granzyme B; HAVCR2: hepatitis A virus cellular receptor 2; LAG3: lymphocyte-activation gene 3; PDCD1: programmed cell death protein 1; STAT5B: signal transducer and activator of transcription 5B; TGFB1: transforming growth factor beta 1.

**Figure 7:**
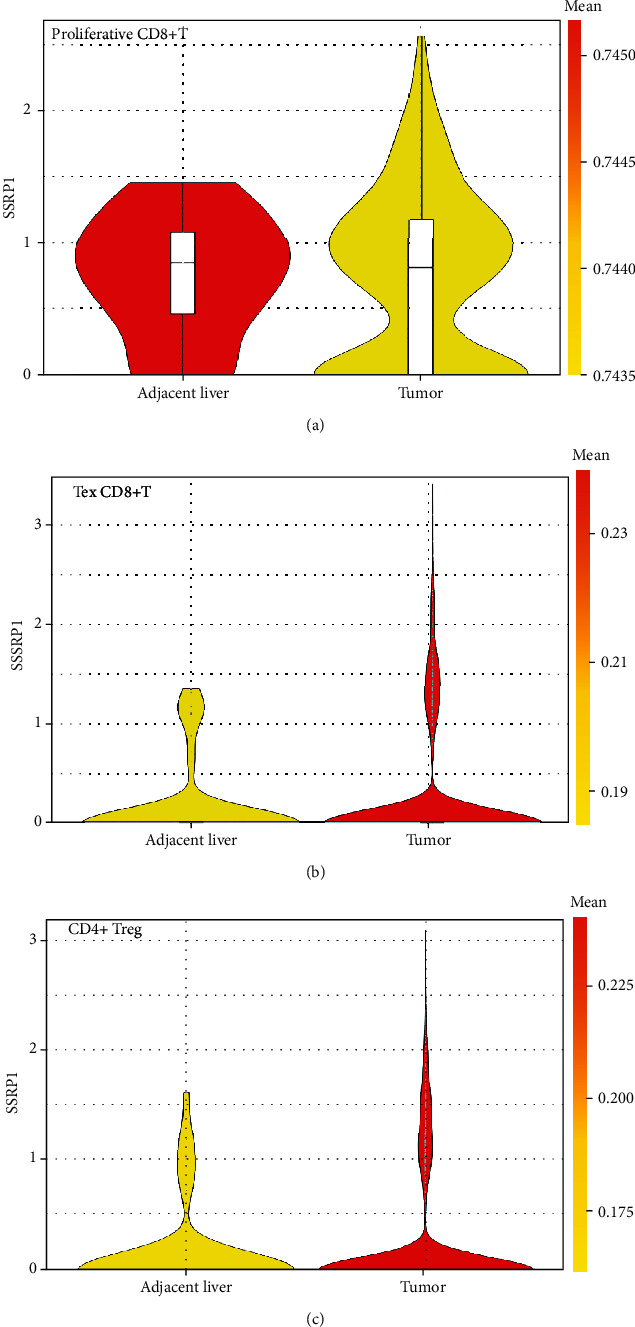
Expression of SSRP1 in different immune cells. (a) Expression of SSRP1 in proliferative CD8^+^ T cells. (b) Expression of SSRP1 in exhausted CD8^+^ T cells. (c) Expression of SSRP1 in Tregs.

It has previously been shown that proliferative CD8^+^ T cells and exhausted CD8^+^ T cells are significantly higher in hepatocellular carcinoma compared to the paraneoplastic tissue (Figure 5). After determining the prognostic value of SSRP1, we performed an analysis of the correlation between SSRP1 and immune infiltration levels in HCC. Elevated SSRP1 was significantly correlated (*p* < 0.05) with Tregs and CD8^+^ T cell infiltration (Figures 6(a) and 6(b)), resulting in increased immune infiltration. At the same time, we analyzed the correlation between the SSRP1 expression and immune marker genes of different immune cells. The results showed that SSRP1 expression levels significantly correlated with most of the immune marker sets of Tregs and exhausted CD8^+^T cells (Figure 6(c)). We then further analyzed the proportion of different CD8^+^T cells in the single cell database and found that exhausted CD8^+^ T cells were significantly higher in the tumor. Therefore, it was reasonable to believe that the increased CD8^+^ T cells were mainly exhausted CD8^+^ T cells. We then went on to further understand the relationship between exhausted CD8^+^ T cells and SSRP1 expression. Interestingly, we found that the SSRP1 expression of tumor exhausted CD8^+^ T cells was lower than that of normal tissues, contrary to that of tumor cells (Figures 7(a)–(c)).

## 4. Discussion

According to the current study, SSRP1 is mainly involved in chromatin-related processes, including DNA replication, repair, and transcription. Furthermore, upregulation of SSRP1 promotes the development of HCC [13], whereas its inhibitor, curaxin, effectively inhibits HCC growth [16]. To better understand the potential function of SSRP1 in HCC and its regulatory network, we performed a bioinformatic analysis of public data to guide future research in HCC. Transcriptome analysis of multiple cohorts including HCCDB and Oncomine, as well as protein expression analysis in HPA, confirmed that SSRP1 mRNA and protein levels were significantly higher in HCC than in the normal liver tissue. Moreover, the high expression of SSRP1 in HCC was significantly associated with shorter overall survival and progression-free survival time. In addition, high levels of the SSRP1 expression were associated with poor prognosis of cellular infiltration of Tregs, CD8^+^ T cells, and B cells. Interestingly, the HR of the SSRP1 high expression was increased when enriched in the above cells. Therefore, our results suggest that SSRP1 deserves further clinical validation as a potential prognostic marker.

To investigate the signaling events controlling the aberrant expression of SSRP1, we tested the coexpression network of SSRP1. The results showed that the functional consequences of SSRP1 mainly include DNA replication, chromosome segregation, and cell cycle G2/M phase transition, while it inhibits acute inflammatory responses, peroxisome organization, and lipid catabolism processes. These findings are consistent with the molecular pathway of HCC carcinogenesis.

Previous studies have shown that Tregs are enriched at the tumor edge, while proliferative CD8^+^ T cells and exhausted CD8^+^ T cells are enriched in the tumor core. Another important aspect of this study is that in HCC, there is a significant positive correlation between SSRP1 expression levels and Tregs as well as CD8+ T cell infiltration levels. The results indicate that SSRP1 has the potential to activate Tregs and induce CD8^+^ T cell exhaustion. The increased expression of SSRP1 was positively correlated with the expression of Treg and T cell exhaustion markers (CCR8, CTLA4, FOXP3, GZMB, HAVCR2, LAG3, PDCD1, STAT5B, and TGFB1) in HCC. Among them, TIM-3 is an important surface protein on exhausted T cells. Taken together, these findings suggest that SSRP1 plays an important role in the recruitment and regulation of immune infiltrating cells in HCC.

Recent studies have provided possible mechanisms that explain why the SSRP1 expression is associated with immune infiltration and poor prognosis. It has been shown in previous studies that SSRP1 inhibitors inhibit NF-*κ*B-dependent transcription in breast cancer [23], non-small-cell lung cancer [24], and glioblastoma [15]. Subsequently, it was shown that 2 weeks after melanoma cell inoculation, live CD8^+^ T cells increased equally in p65- and c-Rel-deficient mice compared to WT littermates; in contrast, the proportion of TIL Tregs was considerably reduced [25]. Therefore, we speculate that SSRP1 may induce CD8^+^ T cell exhaustion via NF-*κ*B.

## 5. Conclusion

To sum up, as a new prognostic marker of liver cancer, SSRP1 promotes the development of HCC by affecting immune cell infiltration and may affect the effect of immunotherapy.

## Figures and Tables

**Table 1:** Clinicopathological characteristics baseline in relation to the SSRP1 expression level in the Kaplan-Meier plotter database.

Clinicopathological characteristics	Liver cancer, overall survival (*n* = 364)			Liver hepatocellular carcinoma, overall survival (*n* = 7462)		
	*N*	Hazard ratio	*p* value	*N*	Hazard ratio	*p* value
AJCC T						
1	181	2.33 (1.07-5.05)	0.028			
2	94	2.96 (1.26-6.95)	<0.01			
3	80	1.71 (0.91-3.22)	0.092			
Vascular invasion						
None	205	2.05 (1.19-3.53)	<0.01			
Macro	93	2.79 (0.84-9.33)	0.082			
RACE						
Asian	158	3.06 (1.69-5.54)	<0.01			
White	184	2.51 (1.37-4.6)	<0.01			
Alcohol consumption						
Yes	117	2.42 (1.27-4.6)	<0.01			
None	205	2.19 (1.35-3.55)	<0.01			
Hepatitis virus						
Yes	153	2.22 (1.15-4.29)	0.015			
None	169	3.19 (1.78-5.7)	<0.01			
CD8^+^T cells						
Enriched				3601	3.05 (1.53-6.09)	<0.01
Decreased				3416	2.48 (1.63-3.78)	<0.01
Tregs						
Enriched				3434	1.75 (1.15-2.66)	<0.01
Decreased				3583	1.53 (0.70-3.34)	0.29
B cell						
Enriched				3763	3.50 (1.52-8.06)	<0.01
Decreased				3254	2.54 (1.69-3.80)	<0.01
Macrophages						
Enriched				3983	2.65 (1.66-4.24)	<0.01
Decreased				3034	2.96 (1.63-5.35)	<0.01

## Data Availability

The analyzed data sets generated during the study are available from public databases or the corresponding author on reasonable request.
